# Acute patients discharged without an established diagnosis: risk of mortality and readmission of nonspecific diagnoses compared to disease-specific diagnoses

**DOI:** 10.1186/s13049-024-01191-4

**Published:** 2024-04-19

**Authors:** Rasmus Gregersen, Marie Villumsen, Katarina Høgh Mottlau, Cathrine Fox Maule, Hanne Nygaard, Jens Henning Rasmussen, Mikkel Bring Christensen, Janne Petersen

**Affiliations:** 1 Department of Emergency Medicine, Copenhagen University Hospital– Bispebjerg and Frederiksberg, Copenhagen, Denmark; 2 Center for Clinical Research and Prevention, Copenhagen University Hospital– Bispebjerg and Frederiksberg, Copenhagen, Denmark; 3 Department of Public Health, Faculty of Health and Medical Sciences, University of Copenhagen, Copenhagen, Denmark; 4 Copenhagen Center for Translational Research, Copenhagen University Hospital– Bispebjerg and Frederiksberg, Copenhagen, Denmark; 5 Department of Clinical Pharmacology, Copenhagen University Hospital– Bispebjerg and Frederiksberg, Copenhagen, Denmark; 6 Department of Clinical Medicine, University of Copenhagen, Copenhagen, Denmark

**Keywords:** Ill-defined diagnoses, Unspecific diagnoses, Diagnostic error, Diagnostically unresolved, Emergency medicine, Acute medicine, Urgent care

## Abstract

**Background:**

Nonspecific discharge diagnoses after acute hospital courses represent patients discharged without an established cause of their complaints. These patients should have a low risk of adverse outcomes as serious conditions should have been ruled out. We aimed to investigate the mortality and readmissions following nonspecific discharge diagnoses compared to disease-specific diagnoses and assessed different nonspecific subgroups.

**Methods:**

Register-based cohort study including hospital courses beginning in emergency departments across 3 regions of Denmark during March 2019–February 2020. We identified nonspecific diagnoses from the R- and Z03-chapter in the ICD-10 classification and excluded injuries, among others—remaining diagnoses were considered disease-specific. Outcomes were 30-day mortality and readmission, the groups were compared by Cox regression hazard ratios (HR), unadjusted and adjusted for socioeconomics, comorbidity, administrative information and laboratory results. We stratified into short (3–<12 h) or lengthier (12–168 h) hospital courses.

**Results:**

We included 192,185 hospital courses where nonspecific discharge diagnoses accounted for 50.7% of short and 25.9% of lengthier discharges. The cumulative risk of mortality for nonspecific vs. disease-specific discharge diagnoses was 0.6% (0.6–0.7%) vs. 0.8% (0.7–0.9%) after short and 1.6% (1.5–1.7%) vs. 2.6% (2.5–2.7%) after lengthier courses with adjusted HRs of 0.97 (0.83–1.13) and 0.94 (0.85–1.05), respectively. The cumulative risk of readmission for nonspecific vs. disease-specific discharge diagnoses was 7.3% (7.1–7.5%) vs. 8.4% (8.2–8.6%) after short and 11.1% (10.8–11.5%) vs. 13.7% (13.4–13.9%) after lengthier courses with adjusted HRs of 0.94 (0.90–0.98) and 0.95 (0.91–0.99), respectively. We identified 50 clinical subgroups of nonspecific diagnoses, of which *Abdominal pain* (*n* = 12,462; 17.1%) and *Chest pain* (*n* = 9,599; 13.1%) were the most frequent. The subgroups described differences in characteristics with mean age 41.9 to 80.8 years and mean length of stay 7.1 to 59.5 h, and outcomes with < 0.2–8.1% risk of 30-day mortality and 3.5–22.6% risk of 30-day readmission.

**Conclusions:**

In unadjusted analyses, nonspecific diagnoses had a lower risk of mortality and readmission than disease-specific diagnoses but had a similar risk after adjustments. We identified 509 clinical subgroups of nonspecific diagnoses with vastly different characteristics and prognosis.

**Supplementary Information:**

The online version contains supplementary material available at 10.1186/s13049-024-01191-4.

## Background

As the main purpose of emergency departments (ED) is to establish and treat diagnoses that need acute treatment, some presentations can await non-acute workup [1, 2]. Therefore, patients can be discharged without an established diagnosis, for instance when symptoms are thought transitory and unrelated to diseases or when the patient can be safely discharged to further diagnostics at general practitioners (GP) or outpatient clinics. According to the International Classification of Diseases 10th revision (ICD-10), patients who are discharged without an established diagnosis should be registered with a nonspecific primary discharge diagnosis [3]. Thereby, nonspecific primary discharge diagnoses represent patients that does not have an established diagnosis at discharge. This practice is frequent, as nonspecific diagnoses are registered for 15–50% of acute hospital discharges in increasing numbers [4–11]. Since serious or life-threatening conditions should have been ruled out and the patient appear clinically stable and fit for discharge, a good short-term prognosis should be expected. However, some studies have reported a 30-day risk of mortality at 0.8-3% and a risk of acute readmissions as high as 30% for patients with nonspecific diagnoses [8–15]. Further, of all deaths within 8 or 30 days after ED discharge, 11–20% have been reported to be preceded by a nonspecific discharge diagnosis [10, 12, 14, 16]. Some have reported a higher occurrence of nonspecific discharge diagnoses after arriving by ambulance, in metropolitan areas, for both young children and the oldest patients, men, and non-Caucasians [4, 11], but the associations are ambiguous [13]. However, direct comparisons of patients discharged with nonspecific and disease-specific diagnoses, adjusting for relevant confounders, have not been conducted.

The aim of this paper was to investigate patient characteristics, mortality and readmissions for patients discharged from acute hospital courses with nonspecific diagnoses and to compare them with patients discharged with disease-specific diagnoses. Further, we aimed to identify clinically homogenous subgroups of nonspecific diagnoses and assess each group’s characteristics and risk of outcomes.

## Methods

We conducted a register-based cohort study including hospital courses beginning in EDs across three regions of Denmark from March 1, 2019, to February 28, 2020.

### Setting

Denmark has a population of 5.8 m, with five organizational regions responsible for running public hospitals and emergency medical services (EMS), among others [17, 18]. Denmark employs a tax-funded, universal healthcare and welfare system, including examination, diagnostics and treatment at public hospitals. Access to acute care requires a referral from GPs, out-of-hours doctors or EMS by pre-hospital physical or telephonic evaluation to limit unnecessary contacts, although self-referrals are treated if they need immediate treatment [2, 17, 18]. Some conditions are brought directly to specialized departments, most notably ST-elevated myocardial infarction, stroke with thrombolysis potential and, in some instances, worsening of a known condition [19]. The Capital Region has a different setup for handling out-of-hours GP services, as these are handled in separate ED tracks rather than at GPs [5, 18]. Patients can typically stay under the EDs responsibility for up to 48 h in emergency wards [19]. 

### Study population

**Fig. 1:**
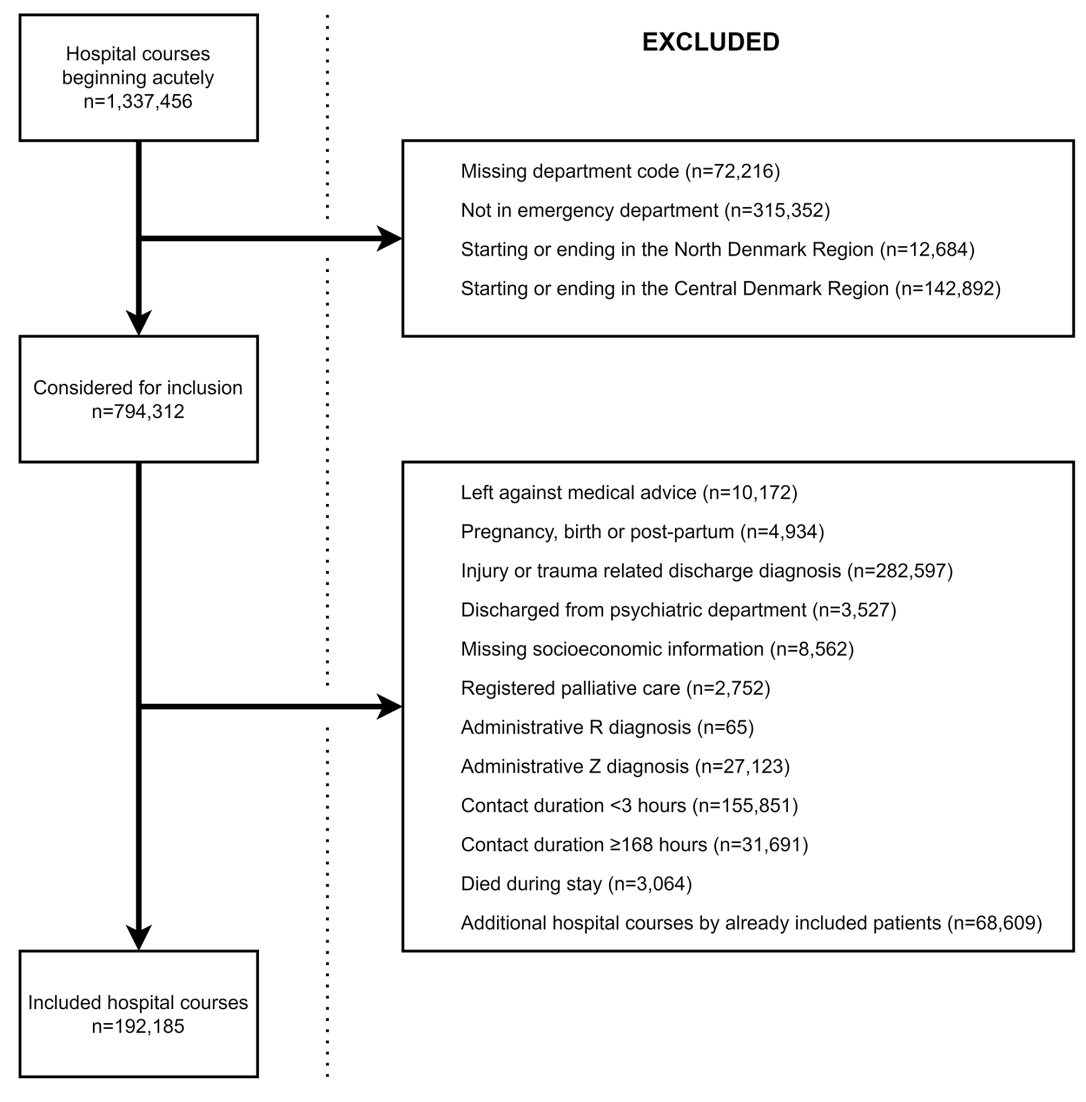
Inclusion and exclusion flow diagram

We included hospital courses beginning in EDs (Supplementary Table S1) by adults (≥ 18 years of age) with a permanent CPR number. The hospital courses could extend into inpatient wards if the patient was transferred for further observation. We did not consider hospital courses starting or ending in the North or Central Denmark Region due to a incomplete data (missing department codes and laboratory information, respectively). We excluded patients who left against medical advice (ICD-10: DZ766*), diagnoses related to pregnancy, birth or the postpartum period (ICD-10: DO*, DZ3*, DZ038O, DZ038M), patients discharged with diagnoses of injury or trauma (as the abundance of patients with minor trauma have a distinctly good prognosis after discharge) [5, 20], discharges from psychiatric departments, missing sociodemographic information, patients with registered palliative care (ICD-10: DZ515* or procedure-code BXB), administrative codes from the R and Z chapters (Supplementary table S2), hospital courses lasting < 3 (to exclude brief out-of-hours consultations) or ≥ 168 h, and mortality during stay or the day of discharge (Fig. 1). If a patient was eligible for inclusion more than once, we only included the first hospital course. In the Danish National Patient Registry (DNPR), there is one contact for each stay or consultation at a specific department. For each contact, a primary diagnosis must be registered along with optional secondary diagnoses. Hospital courses were formed by combining individual contacts within 4 h, including both somatic and psychiatric contacts using the %DNPR_contact_combine SAS-macro (Gregersen et al., unpublished).

### Outcomes and exposure

The primary outcomes were post-discharge 30-day mortality and 30-day readmission. A readmission was defined as a new hospital course initiated acutely and lasting for ≥ 12 h. Among secondary outcomes, we examined death causes (natural: old age or disease; unnatural: trauma, violence or suicide), the primary discharge diagnosis at readmissions (nonspecific or disease-specific), 7-day mortality and 7-day readmission.

The exposure was whether the primary discharge diagnosis (the final primary diagnosis at the end of the full hospital course) was nonspecific or disease-specific. Per ICD-10 guidelines, a diagnosis code from the R chapter should only be registered when there is no established or likely diagnosis [3]. Likewise, the Z03 chapter is used for patients observed and examined under suspicion of certain conditions that ended up being ruled out or not fully established [3]. Three medical doctors (authors RG, KHM and MBC) reviewed all registered primary diagnoses from the R and Z chapters and separated them into disease-specific, nonspecific or administrative groups in an ED setting. Disagreements were settled by discussion. After the exclusion of codes defining injuries, pregnancy-related and administrative diagnoses, disease-specific codes from the R and Z chapter were grouped with the remaining ICD-10 chapters to form the disease-specific group and the remaining R and Z03 codes were considered nonspecific (Supplementary table S2).

To further explore if different nonspecific discharge symptoms have dissimilar characteristics or risks, the same authors reviewed the nonspecific diagnoses and grouped these into clinically homogenous subgroups (e.g., *Abdominal pain*, *Vertigo*, etc., Supplementary Table S2).

### Covariates

We examined sociodemographics, comorbidity, administrative information and laboratory results [5]. Sociodemographics were registered at the index date or the end of the previous calendar year and included age, sex, educational level, personal disposable income in quintiles compared to the general population, source of income, civil status, co-habitation and a combined measure of immigration and country of origin. Comorbidity was assessed by the M3 Comorbidity Index from primary and secondary in- or outpatient hospital diagnoses until 10 years prior to the primary hospital course [21]. Administrative information included time and day of discharge and arrival as well as length of stay. We identified the 16 most frequently used routine acute blood tests (hemoglobin, leukocytes, thrombocytes, prothrombin international normalized ratio [INR], sodium, potassium, creatinine, estimated glomerular filtration rate [eGFR], albumin, bilirubin, alanine transaminase [ALAT], alkaline phosphatase [ASAT], amylase, C-reactive protein, glucose and lactate dehydrogenase) during the hospital course ± 6 h. Among these, we assessed whether at least 5 different results were analyzed (as a proxy of whether a full blood analysis was conducted) and the number of tests outside the registered reference range, using the latest test if repeated tests were available [22]. 

### Data sources

We used the Bispebjerg Acute Cohort [5] containing information from the DNPR [23], the Register of Laboratory Results for Research (RLRR) [24], the Central Person Register (CPR) [25], the Danish Register of Causes of Death [26], the Danish Education Register [27] and the Income Statistics Register [28], including data from 2003 and onwards. Linkage was done through the unique CPR number [17]. The DNPR includes all contacts to public hospitals in Denmark, classified as either acute or elective.

### Statistical methods

Continuous baseline characteristics were presented by median, first and third quartiles (Q1;Q3) and categorial variables by number and percentage (%). The outcomes were analyzed as cumulative incidences with a 95% confidence interval (CI). Unadjusted and adjusted Cox regression analyses were presented as hazard ratios (HR). We adjusted for demographics (age and sex), socioeconomics, comorbidity (M3 score and a binary indicator of any M3 cancer diagnosis), administrative information and laboratory results (binary indicator of laboratory results and number of abnormal results). Death was considered a competing risk of readmission. Analyzes and results were stratified by total length-of-stay as either short (3–12 h) or lengthier (12–168 h) hospital courses. Assumption of proportional hazards was assessed by inspecting the cumulative sums of Martingale-based and Schoenfeld residuals and testing the significance of an interaction between the exposure and time. For each of the identified clinical subgroups among patients with nonspecific diagnoses, we assessed age, sex, M3 score, length-of-stay and 30-day risk of mortality and readmission. Due to rules of data protection, groups with < 5 events were censored. Reporting adhered to RECORD guidelines [29]. 

## Results

We included 192,185 hospital courses of unique patients (Fig. 1). Of these, 93,301 (48.5%) had a short hospital course (< 12 h). Nonspecific discharge diagnoses were registered for 50.7% of short and 25.9% of lengthier hospital courses (Table 1). Patients discharged with nonspecific diagnoses after short hospital courses were more often female and had blood samples analyzed more frequently but were comparable in age, comorbidity and socioeconomics with patients with disease-specific diagnoses. Patients with lengthier courses and nonspecific discharge diagnoses were slightly younger, more often female, more often in employment or studying, more often unmarried, less frequently living alone, were less often of Danish origin, had a notably shorter length-of-stay (median 25.1 vs. 50.8 h) and fewer abnormal blood test results compared to patients with disease-specific diagnoses.

**Table 1:** Patient characteristics, administrative and biochemical information for included patients, stratified by total contact length and nonspecific or disease-specific discharge diagnosis

		Short hospital course		Lengthier hospital course	
		**Nonspecific diagnoses** *n* = 47,308 (50.7%)	**Disease-specific diagnoses** *n* = 45,993 (49.3%)	**Nonspecific diagnoses** *n* = 25,642 (25.9%)	**Disease-specific diagnoses** *n* = 73,242 (74.1%)
Age	Median (Q1-Q3)	56.0 (38.0–72.0)	56.0 (39.0–72.0)	66.0 (47.0–78.0)	69.0 (53.0–79.0)
Sex	Female	54.2%	50.5%	52.5%	49.1%
M3 Score	0	36.9%	36.9%	26.9%	23.1%
	0 to < 1	46.2%	45.2%	47.6%	46.1%
	1 to < 2	12.0%	12.7%	17.2%	20.7%
	≥ 2	4.9%	5.2%	8.3%	10.1%
Educational level	Missing or no primary education	1.9%	1.9%	2.0%	2.2%
	Primary or lower secondary (ISCED 0–2)	32.1%	31.4%	36.2%	36.7%
	Upper secondary or post-secondary, non-tertiary (ISCED 3–4)	40.3%	40.7%	39.9%	40.1%
	Short-cycle tertiary or above (ISCED 5–8)	25.7%	26.0%	21.9%	21.0%
Source of income	Active employment or student	49.5%	49.7%	35.7%	30.8%
	Unemployed, sick leave, absence allowance etc.	9.6%	9.5%	8.2%	7.1%
	Disability pensioner	7.5%	7.1%	8.2%	7.9%
	Old age pensioner and early retirement benefit	33.5%	33.6%	47.9%	54.1%
Civil status	Married	44.4%	43.9%	44.2%	44.7%
	Unmarried	30.1%	30.5%	23.2%	20.7%
	Divorced	15.5%	15.7%	16.7%	16.8%
	Widowed	10.0%	9.9%	16.0%	17.8%
Co-habitation	Single, living alone	33.0%	33.4%	38.6%	40.9%
	Single, living with at least one other person	9.5%	10.1%	7.3%	6.9%
	Couples, living with at least one other person	57.5%	56.5%	54.1%	52.1%
Origin	Danish origin	83.3%	84.6%	88.9%	91.0%
	Western origin: Immigrant or descendant of immigrants	4.3%	4.2%	3.4%	3.3%
	Non-western origin: Immigrant or descendant	12.4%	11.2%	7.7%	5.7%
Length-of-stay	Median (Q1-Q3)	5.2 (4.0-7.1)	5.1 (3.9–7.1)	25.2 (18.1–49.5)	50.9 (24.7–96.0)
Laboratory results	No or few (< 4) blood tests	23.2%	32.0%	8.7%	8.3%
	Blood tests, 0–1 abnormal result	34.6%	23.0%	29.4%	15.8%
	Blood tests, 2–3 abnormal results	28.8%	27.4%	33.6%	30.8%
	Blood tests, 4 + abnormal results	13.5%	17.7%	28.3%	45.1%

*Abbreviations* ISCED = International Standard Classification of Education, M3 Score = M3 comorbidity index score, Q1 = first quartile, Q3 = third quartile

The primary discharge diagnosis at readmissions was more often nonspecific again after an initial nonspecific discharge compared to a after an initial disease-specific discharge at 33.7% vs. 16.2% (*p* < 0.001).

### Short hospital courses

**Fig. 2:**
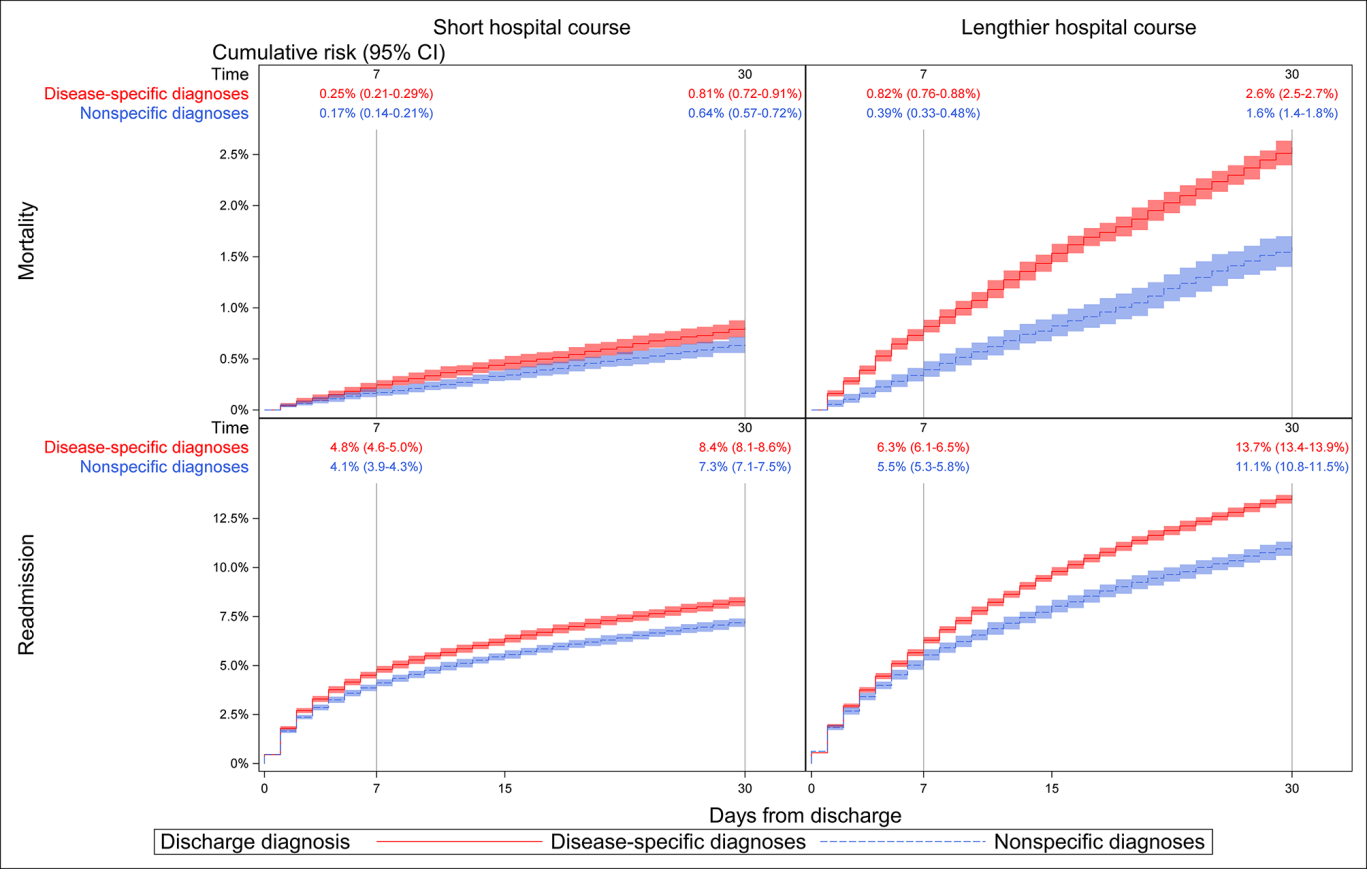
Cumulative incidence plots of mortality and readmission for patients discharged with nonspecific and disease-specific diagnoses up to 30 days from discharge, stratified into short and lengthier hospital courses

After short hospital courses, discharges with nonspecific diagnoses preceded 45.0% of all mortality and 47.3% of readmissions within 30 days (Table 2). The cumulative risk of mortality was 0.6% (0.6–0.7%) for nonspecific and 0.8% (0.7–0.9%) for disease-specific discharge diagnoses (Fig. 2) with an unadjusted HR of 0.79 (0.68–0.92). The cumulative risk of readmission was 7.3% (7.1–7.5%) for patients with nonspecific and 8.4% (8.2–8.6%) for patients with disease-specific discharge diagnoses with an unadjusted HR of 0.87 (0.83–0.91). Adjustments diminished the difference in mortality HR of 0.97 (0.83–1.13) and readmission HR of 0.94 (0.90–0.98) (see Supplementary Table S3 for stepwise adjustments).

**Table 2:** Unadjusted and adjusted Cox regression hazard ratios of mortality and acute readmission, respectively, within 30 and 7 days for patients discharged with nonspecific diagnoses compared to disease-specific diagnoses, stratified into short and lengthier hospital courses

			Mortality		Readmission^†^	
30-day outcomes			Nonspecific discharge diagnosis	Disease-specific discharge diagnosis	Nonspecific discharge diagnosis	Disease-specific discharge diagnosis
**Short hospital courses** **(3 - <12 h)**	Number of events (%)		304 (45.0%)	372 (55.0%)	3460 (47.3%)	3856 (52.7%)
	HR	Unadjusted	0.79 (0.68–0.92)	(ref)	0.87 (0.83–0.91)	(ref)
		Adjusted*	0.97 (0.83–1.13)	(ref)	0.94 (0.90–0.98)	(ref)
**Lengthier hospital courses** **(12–168 h)**	Number of events (%)		409 (17.8%)	1885 (82.2%)	2859 (22.2%)	10,036 (77.8%)
	HR	Unadjusted	0.62 (0.55–0.68)	(ref)	0.80 (0.77–0.83)	(ref)
		Adjusted*	0.94 (0.85–1.05)	(ref)	0.95 (0.91–0.99)	(ref)
**7-day outcomes**						
**Short hospital courses** **(3 - <12 h)**	HR	Unadjusted	0.70 (0.52–0.93)	(ref)	0.85 (0.80–0.91)	(ref)
		Adjusted*	0.84 (0.63–1.12)**	(ref)	0.92 (0.86–0.98)	(ref)
**Lengthier hospital courses** **(12–168 h)**	HR	Unadjusted	0.48 (0.39–0.59)	(ref)	0.88 (0.83–0.93)	(ref)
		Adjusted*	0.72 (0.57–0.88)	(ref)	0.97 (0.91–1.03)	(ref)

*Abbreviations* HR = hazard ratio, CI = confidence interval

† Death before readmission was considered a competing event

* Adjusted for demographics (age and sex), socioeconomics (educational level, employment, civil status, cohabitation status, income, immigration status and country of origin), comorbidity (M3 comorbidity score and M3 cancer diagnosis), administrative information (time and day of arrival, time and day of discharge, length of stay) and laboratory information (whether at least 5 blood tests were analyzed among the 16 most frequent analyses and number of abnormal results among these)

** Not adjusted for all socioeconomic variables and no administrative variables due to limited number of events

### Lengthier hospital courses

After lengthier hospital courses, discharges with nonspecific diagnoses preceded 17.8% of all mortality and 22.2% of readmissions within 30 days (Table 2). The cumulative risk of mortality was 1.6% (1.5–1.7%) for nonspecific and 2.6% (2.5–2.7%) for disease-specific discharge diagnoses (Fig. 2) with an unadjusted HR of 0.62 (0.55–0.68). The HR increased and became insignificant after adjustment at 0.94 (0.85–1.05). The cumulative risk of readmission was 11.1% (10.8–11.5%) for patients with nonspecific and 13.7% (13.4–13.9%) for patients with disease-specific discharge diagnoses with an unadjusted HR of 0.80 (0.77–0.83). The HR increased but remained significant after adjustments at 0.95 (0.91–0.99).

### Clinical subgroups

Among the 72,950 patients with a nonspecific discharge diagnosis, 337 different primary ICD-10 codes were registered. After reviewing them, we formed 38 clinical groups from the R chapter and 12 groups from the Z03 chapter. The clinical subgroups varied notably in size (*n* = 35 to *n* = 12,462), mean age (41.9 to 80.8 years) and mean length of stay (7.1 to 59.5 h), among others (Supplementary Table S3). *Abdominal pain* and *Chest pain* were the biggest groups and accounted for 17.1% and 13.1% of all patients with nonspecific diagnoses, respectively. Among the 20 largest groups, the risk of mortality within 30 days was below the censored threshold (< 5 events) for some groups (*Vertigo*, *Headache* and *Palpitations)*, and else differed from 0.2% (0.1–0.3%) for *Chest pain* to 8.1% (6.5–10.2%) for *Observation for suspected cancer* (Table 3). The risk of 30-day readmission was lowest for *Palpitations* at 3.5% (2.7–4.7%) and highest for *Observation for suspected cancer* at 22.6% (20.2–25.3%). In the groups not among the 20 most frequent, some had a notably high risk of outcomes: *Ascites* (*n* = 139; 15.0% 30-day mortality; 33.5% 30-day readmission), *Icterus* (*n* = 126; 7.1% 30-day mortality; 27.6% 30-day readmission), *Chronic pain* (*n* = 120; 17.4% 30-day readmission), and *Abnormal weight loss and cachexia* (*n* = 72; 9.6% 30-day mortality; 20.6% 30-day readmission) (Supplementary table S3). Excluding these four aforementioned smaller groups with high risk did not alter the overall risk estimates.

**Table 3:** Basic characteristics, risk of mortality and readmission for all patients discharged with nonspecific diagnoses for the 20 most frequent clinical subgroups

Group	N	Age, mean	Female, %	M3 Score, mean	LOS (hours), mean	Risk of mortality within 30 days, % (95% CI)	Risk of readmission within 30 days, % (95% CI)
All R and Z03	72,950	57.3	53.6%	0.55	18	1.0% (0.9–1.1%)	8.6% (8.4–8.9%)
All R	58,924	56.9	54.1%	0.55	17.6	0.9% (0.9-1.0%)	8.8% (8.6-9.0%)
Chest pain	9599	55.1	50.8%	0.45	11.1	0.2% (0.1–0.3%)	4.5% (4.2–4.9%)
Palpitations	1299	50.9	63.8%	0.33	7.8	Censored, few events	3.5% (2.7–4.7%)
Abnormal breathing	3788	64.2	55.9%	0.89	22.3	2.7% (2.3–3.3%)	12.5% (11.5–13.6%)
Epistaxis and oropharyngeal bleeding	1160	69.6	45.6%	0.73	11.2	1.2% (0.7-2.0%)	10.6% (9.2–12.3%)
Abdominal pain	12,462	46.9	64.3%	0.41	15.2	0.5% (0.4–0.6%)	9.7% (9.2–10.1%)
Nausea or vomiting	998	56.3	61.1%	0.68	21.7	1.7% (1.1–2.7%)	13.7% (11.6–16.1%)
Symptoms and signs involving the nervous and musculoskeletal systems	2421	54.9	58.3%	0.49	26.7	0.2% (0.1–0.6%)	7.4% (6.4–8.5%)
Tendency to fall	958	80.8	54.0%	1.03	48.3	2.3% (1.5–3.5%)	16.0% (14.1–18.2%)
Seizures	1122	51.7	39.3%	0.79	22.1	1.1% (0.6–1.8%)	9.1% (7.8–10.5%)
Vertigo	2921	64.3	59.3%	0.48	18.3	Censored, few events	6.0% (5.2-7.0%)
Headache, unspecified	2578	47.6	62.6%	0.34	13.7	Censored, few events	7.5% (6.5–8.7%)
Symptoms regarding urination and urinary tract	2495	71.6	15.3%	0.77	26.2	2.1% (1.6–2.8%)	16.1% (14.7–17.7%)
Malaise or fatigue	2214	63.1	56.1%	0.64	10.8	1.4% (1.0-1.9%)	8.4% (7.5–9.5%)
Fainting	4708	62	49.2%	0.52	20.5	0.4% (0.3–0.6%)	5.9% (5.3–6.5%)
Fever	1046	53.9	43.0%	0.64	32.1	1.3% (0.8–2.4%)	14.6% (12.9–16.7%)
Acute pain, unspecified	3460	56.5	59.0%	0.51	10.6	0.8% (0.6–1.2%)	7.8% (6.9–8.8%)
All Z03	14,026	59.5	51.3%	0.56	19.7	1.2% (1.0-1.4%)	8.1% (7.6–8.6%)
Observation for suspected myocardial infarction	2656	61.7	47.6%	0.58	21.1	0.3% (0.1–0.5%)	5.3% (4.6–6.1%)
Observation for suspected cancer	833	69.3	43.0%	0.6	58.8	8.1% (6.5–10.2%)	22.6% (20.2–25.3%)
Observation for concussion	1526	59.3	49.4%	0.61	10.2	1.0% (0.7–1.7%)	6.7% (5.6-8.0%)
Observation for unspecified disease or condition	4973	56.5	53.3%	0.55	12.3	1.0% (0.8–1.3%)	8.4% (7.6–9.3%)

Cells with < 5 events are censored

*Abbreviations* CI = confidence interval, LOS = length-of-stay

## Discussion

In this cohort study, we identified a reduced risk of 30-day mortality and readmission for patients with a nonspecific primary discharge diagnosis compared with patients discharged with a disease-specific primary diagnosis after both short and lengthier hospital courses beginning in the ED. However, after adjustments for confounders, this reduced risk of mortality turned insignificant and diminished notably for readmissions. Nonspecific discharge diagnoses were frequent and accounted for almost half of the adverse outcomes after short hospital courses and about one-fifth after lengthier courses. Patients with nonspecific discharge diagnoses were a heterogenous group from which we identified 50 different clinical subgroups describing a wide span of causes, patient characteristics and risks of both death and readmission.

Clinically, patients with nonspecific diagnoses should represent patients that are discharged without an established diagnosis: either believed without acute diseases or with diseases that do not need to be fully established in an acute setting [3]. With these results, it is reasonable to question if the prognosis is as favorable as should be expected, given the clinical context. This does not imply that all patients discharged without an established diagnosis are without diseases—but those awaiting further diagnostics should have a solid plan for follow-up without dying or needing acute readmissions. It is possible that some patients are discharged with the purpose of dying at home, thus full diagnostics are not conducted, resulting in a nonspecific diagnosis and expected poor prognosis. We sought to mitigate this by excluding patients in palliative care. While we demonstrated that patients with nonspecific diagnoses are a diverse group representing different clinical subgroups, the same is true for patients with disease-specific diagnoses, comprising a wide range of patient characteristics, clinical conditions and prognoses. It is important to remember that these patients are treated as needed, given their disease-specific diagnosis. Thereby, these comparisons mainly highlight that patients who are discharged with nonspecific diagnoses should be treated with almost the same caution of adverse outcomes as patients discharged with and treated for established diseases.

A cause for the adverse outcomes could be diagnostic errors, where diseases are not recognized during the acute contact and are, therefore, missed or delayed [30–33]. Diagnostic errors can occur from unacknowledged cognitive biases, causing impaired decision-making during the diagnostic process [33–35]. Emergency physicians are prone to diagnostic errors due to the sheer amount of patients, complex presentations and the need for timely and accurate diagnoses to avoid overcrowding [31, 36]. Diagnostic errors among patients transferred from emergency departments (ED) are frequent but remain unexplored after discharge [37]. We only considered the discharge diagnoses of a completed hospital course, as those display that the patient has been deemed fit for discharge.

Our findings on mortality and readmission are largely in concordance with other studies. A study on non-trauma ED visits in the North Denmark Region between 2014 and 2016 presented a high risk of 30-day mortality for R diagnoses at 0.8% after short stays (4–24 h) and 1.1% after very short stays (< 4 h) [10]. Further, a UK study on 6760 elderly patients discharged from internal medicine and geriatric wards revealed a 0.9% and 1.8% risk of 30-day post-discharge mortality, and 10.5% and 9.7% risk of 30-day readmission for R diagnoses and other codes, respectively [13]. Our study adds valuable information that most of the difference in risk of mortality and readmission is explained by differences in patient characteristics, administrative information and biochemical analyses. A study from Iceland investigated 30,221 ED discharges and found a reduced 30-day mortality for R diagnoses compared to other discharge diagnoses with an HR of 0.60 adjusted for age and sex [16], slightly lower than this study’s HR. However, as unspecific diagnoses are found in both the Z03 and R chapters, no studies have focused on the full effect of nonspecific diagnoses at discharge.

Acute readmissions can have different causes, such as exacerbation of symptoms, uncertainty, anxiety or new, unrelated symptoms. Readmissions might also occur as a natural part of a diagnostic work-up, where patients with mild, nonspecific symptoms are discharged and instructed to return if the condition worsens. To disregard brief re-evaluations, we only considered acute readmissions with a total length of stay of at least 12 h. Of the patients discharged with nonspecific diagnoses and experiencing acute readmission, 33.7% were re-assigned a nonspecific diagnosis at the readmission and thereby remained diagnostically unclear. Another study found an increased risk of obtaining an R diagnosis at readmission with HR of 1.6 (1.3–1.8) after discharge with a primary R diagnosis compared to other diagnoses [13]. 

In our study, we discovered a very low risk of 30-day mortality and readmission for symptoms primarily considered related to the cardiovascular system, such as *Chest pain, Fainting*, *Observation for suspected myocardial infarction* and *Palpitations*, indicating good rule-out tests such as ECG and high-sensitivity troponins available to supplement good history taking and clinical assessment. A previous study from the US has described an 8.6% risk of 7-day revisits among patients discharged with nonspecific chest pain [38]. However, only 1.2–2.7% of all their patients needed inpatient care (comparable with our 30-day readmission rate of 4.5%) and < 0.5% had acute coronary syndrome. In a Finnish study, 6.1% of all ED visits were discharged with nonspecific abdominal pain, of which 3.0% had a revisit within the first 48 h [39]. This is comparable to our findings, where nonspecific abdominal pain was the largest group, constituting 6.5% of all included hospital courses (17% of those with nonspecific diagnoses) and entailed a low risk of mortality but a notable risk of acute readmission at 9.7% within 30 days. Further, they also found that two-thirds received a new nonspecific diagnosis at the revisit and immediate surgical treatment was only needed for < 0.1% [39]. 

### Limitations

Differences between countries, most importantly differences in mandatory referral, GP accessibility and health care financial models, might cause differences in ED populations and affect external validity and generalization [2]. However, the mean age of patients with lengthier hospital courses in this study was comparable to those admitted from EDs in an American study and the 7-day mortality for patients discharged from the ED was comparable to the 7-day mortality of shorter hospital courses in this present study, considering we excluded injuries [40]. With this study, we developed rather strict inclusion and exclusion criteria to mimic a “normal” non-trauma ED population. Thereby, our results appear comparable to Western countries. Other strengths of the study were the unselected study population, large sample size and relevant variables for adjustments. Further, we conducted the study before SARS-CoV-19 affected the workflow of EDs.

For this study, we assumed that the nonspecific diagnoses have been registered in concordance with the registration guidelines and represent patients that are discharged without an established diagnosis. The validity of R and/or Z03 discharge codes has not been investigated, but data from the DNPR are generally considered of high validity [23]. However, there is a risk of misregistration—both nonspecific diagnoses that should rather have been coded as specific diseases and specific diseases that was not sufficiently supported and should have been coded as a nonspecific diagnosis. Also, it is uncertain whether the adverse outcomes are related to the primary discharge and with increasing follow-up duration, there is an increasing risk that the adverse events are unrelated. However, sensitivity analyses of 7-day outcomes did not alter the results substantially.

## Conclusions

The risk of short-term mortality and readmission for patients with nonspecific diagnoses is low in absolute numbers, but these patients are not to be disregarded as risk-free patients. When adjusting for confounders, the risk is comparable to that of patients with disease-specific diagnoses. Our findings are relevant to clinicians discharging adult ED or acute inpatients in a broad range of clinical specialties. Due to the frequency and risk of nonspecific diagnoses, they constitute a relevant focus for improving post-discharge outcomes for patients with acute hospital needs. Identifying subgroups revealed disparities in characteristics and risks which might be used in risk stratification going forward.

### Electronic supplementary material

Below is the link to the electronic supplementary material.

Supplementary Material 1

Supplementary Material 2

Supplementary Material 3

Supplementary Material 4

## Data Availability

Due to the rules of protection of individual’s data from Statistics Denmark, it is not possible to share the data in any raw or anonymized form. Danish research institutions can obtain permission to access the data on equal terms.

## Declarations

### Meetings

Some preliminary results have been presented at the Society of Acute Medicine Spring Conference, Copenhagen 2023 as an ePoster and at the International Conference on Emergency Medicine, Amsterdam 2023 as an oral presentation.

## Abbreviations

*: including subgroups
CPR: Central person register
DNPR: Danish National Patient Register
ED: Emergency department
EMS: Emergency medical services
GP: General physician
HR: Hazard ratio
ICD-10: International Classification of Diseases, 10th revision
RLRR: Register of Laboratory Results for Research

## References

[1] Morganti K, Bauhoff S, Blanchard J, Abir M, Iyer N, Smith A et al. The Evolving Role of Emergency Departments in the United States. Rand Heal Q. 2013;3(2). 10.7249/rhq-03-02-03 PMC4945168 28083290

[2] Baier N, Geissler A, Bech M, Bernstein D, Cowling TE, Jackson T (2019). Emergency and urgent care systems in Australia, Denmark, England, France, Germany and the Netherlands– analyzing organization, payment and reforms. Health Policy (New York).

[3] World Health Organization (WHO). International statistical classification of diseases and related health problems, 10th Revision (ICD-10): Volume 1, Tabular List. Fifth edition. [Internet]. Vol. 1, World Health Organization. 2016. Available from: https://apps.who.int/iris/handle/10665/246208.

[4] Søvsø MB, Huibers L, Bech BH, Christensen HC, Christensen MB, Christensen EF (2020). Acute care pathways for patients calling the out-of-hours services. BMC Health Serv Res.

[5] Gregersen R, Maule CF, Bak-Jensen HH, Linneberg A, Nielsen OW, Thomsen SF (2022). Profiling Bispebjerg Acute Cohort: database formation, Acute Contact characteristics of a Metropolitan Hospital, and comparisons to Urban and Rural hospitals in Denmark. Clin Epidemiol.

[6] Fløjstrup M, Bogh SB, Henriksen DP, Bech M, Johnsen SP, Brabrand M. Increasing emergency hospital activity in Denmark, 2005–2016: a nationwide descriptive study. BMJ Open. 2020;10(2). 10.1136/bmjopen-2019-031409 PMC7045230 32051299

[7] Christensen EF, Bendtsen MD, Larsen TM, Jensen FB, Lindskou TA, Holdgaard HO et al. Trends in diagnostic patterns and mortality in emergency ambulance service patients in 2007–2014: a population-based cohort study from the North Denmark Region. BMJ Open. 2017;7(8). 10.1136/bmjopen-2016-014508 PMC5724206 28827233

[8] Klinge M, Aasbrenn M, Aasbrenn M, Öztürk B, Christiansen CF, Suetta C (2020). Readmission of older acutely admitted medical patients after short-term admissions in Denmark: a nationwide cohort study. BMC Geriatr.

[9] Søvsø MB, Hermansen SB, Færk E, Lindskou TA, Ludwig M, Møller JM (2018). Diagnosis and mortality of emergency department patients in the North Denmark region. BMC Health Serv Res.

[10] Al-Mashat H, Lindskou TA, Møller JM, Ludwig M, Christensen EF, Søvsø MB (2022). Assessed and discharged– diagnosis, mortality and revisits in short-term emergency department contacts. BMC Health Serv Res.

[11] Wen LS, Espinola JA, Mosowsky JM, Camargo CA (2015). Do emergency department patients receive a pathological diagnosis? A nationally-representative sample. West J Emerg Med.

[12] Nielsen FV, Nielsen MR, Amstrup J, Lorenzen IL, Kløjgaard TA, Færk E (2020). Non-specific diagnoses are frequent in patients hospitalized after calling 112 and their mortality is high - A register-based Danish cohort study. Scand J Trauma Resusc Emerg Med.

[13] Walsh B, Roberts HC, Nicholls PG (2011). Features and outcomes of unplanned hospital admissions of older people due to ill-defined (R-coded) conditions: retrospective analysis of hospital admissions data in England. BMC Geriatr.

[14] Christensen EF, Larsen TM, Jensen FB, Bendtsen MD, Hansen PA, Johnsen SP (2016). Diagnosis and mortality in prehospital emergency patients transported to hospital: a population-based and registry-based cohort study. BMJ Open.

[15] Hansen KM, Nielsen H, Vest-Hansen B, Møllekær A, Thomsen RW, Mølgaard O (2017). Readmission and mortality in patients discharged with a diagnosis of medical observation and evaluation (Z03*-codes) from an acute admission unit in Denmark: a prospective cohort study. BMC Health Serv Res.

[16] Gunnarsdottir OS, Rafnsson V (2008). Death within 8 days after discharge to home from the emergency department. Eur J Public Health.

[17] Schmidt M, Schmidt SAJ, Adelborg K, Sundbøll J, Laugesen K, Ehrenstein V (2019). The Danish health care system and epidemiological research: from health care contacts to database records. Clin Epidemiol.

[18] Lindskou TA, Mikkelsen S, Christensen EF, Hansen PA, Jørgensen G, Hendriksen OM (2019). The Danish prehospital emergency healthcare system and research possibilities. Scand J Trauma Resusc Emerg Med.

[19] Danske Regioner [Danish Regions], Sundhedsstyrelsen [The Danish Health Authority], Sundheds- og Ældreministeriet [The Ministry of Health]. De danske akutmodtagelser - status 2016 [The Danish Emergency Departments - status 2016] [Internet]. 2016. Available from: https://www.ft.dk/samling/20161/almdel/SUU/bilag/121/1706332.pdf.

[20] Vest-Hansen B, Riis AH, Sørensen HT, Christiansen CF (2014). Acute admissions to medical departments in Denmark: diagnoses and patient characteristics. Eur J Intern Med.

[21] Stanley J, Sarfati D (2017). The new measuring multimorbidity index predicted mortality better than Charlson and Elixhauser indices among the general population. J Clin Epidemiol.

[22] Klausen HH, Petersen J, Bandholm T, Juul-Larsen HG, Tavenier J, Eugen-Olsen J (2017). Association between routine laboratory tests and long-term mortality among acutely admitted older medical patients: a cohort study. BMC Geriatr.

[23] Schmidt M, Schmidt SAJ, Sandegaard JL, Ehrenstein V, Pedersen L, Sørensen HT (2015). The Danish National patient registry: a review of content, data quality, and research potential. Clin Epidemiol.

[24] Arendt JFH, Hansen AT, Ladefoged SA, Sørensen HT, Pedersen L, Adelborg K (2020). Existing data sources in clinical epidemiology: Laboratory information system databases in Denmark. Clin Epidemiol.

[25] Schmidt M, Pedersen L, Sørensen HT (2014). The Danish Civil Registration System as a tool in epidemiology. Eur J Epidemiol.

[26] Helweg-Larsen K (2011). The Danish register of causes of death. Scand J Public Health.

[27] Jensen VM, Rasmussen AW (2011). Danish education registers. Scand J Public Health.

[28] Baadsgaard M, Quitzau J (2011). Danish registers on personal income and transfer payments. Scand J Public Health.

[29] Benchimol EI, Smeeth L, Guttmann A, Harron K, Moher D, Peteresen I (2015). The REporting of studies conducted using Observational routinely-collected health data (RECORD) Statement. PLoS Med.

[30] Newman-Toker DE, Pronovost PJ (2009). Diagnostic errors the next frontier for patient safety. JAMA - J Am Med Assoc.

[31] Kachalia A, Gandhi TK, Puopolo AL, Yoon C, Thomas EJ, Griffey R (2007). Missed and delayed diagnoses in the Emergency Department: a study of closed Malpractice Claims from 4 liability insurers. Ann Emerg Med.

[32] Graber M, Gordon R, Franklin N (2002). Reducing diagnostic errors in medicine: what’s the goal?. Acad Med.

[33] Hussain F, Cooper A, Carson-Stevens A, Donaldson L, Hibbert P, Hughes T (2019). Diagnostic error in the emergency department: learning from national patient safety incident report analysis. BMC Emerg Med.

[34] Croskerry P (2003). The importance of cognitive errors in diagnosis and strategies to minimize them. Acad Med.

[35] Kunitomo K, Harada T, Watari T (2022). Cognitive biases encountered by physicians in the emergency room. BMC Emerg Med.

[36] Amaniyan S, Faldaas BO, Logan PA, Vaismoradi M (2020). Learning from Patient Safety incidents in the Emergency Department: a systematic review. J Emerg Med.

[37] Chellis M, Olson JE, Augustine J, Hamilton GC (2001). Evaluation of missed diagnoses for patients admitted from the emergency department. Acad Emerg Med.

[38] Martsolf GR, Martsolf GR, Nuckols TK, Nuckols TK, Fingar KR, Barrett ML (2020). Nonspecific chest pain and hospital revisits within 7 days of care: variation across emergency department, observation and inpatient visits. BMC Health Serv Res.

[39] Saaristo L, Saaristo L, Ukkonen MT, Ukkonen MT, Laukkarinen JM, Laukkarinen JM (2020). The rate of short-term revisits after diagnosis of non-specific abdominal pain is similar for surgeons and emergency physicians - results from a single tertiary hospital emergency department. Scand J Trauma Resusc Emerg Med.

[40] Obermeyer Z, Cohn B, Wilson M, Jena AB, Cutler DM. Early death after discharge from emergency departments: analysis of national US insurance claims data. BMJ. 2017;356. 10.1136/bmj.j239 PMC6168034 28148486

