# Supplementary material for: Acute patients discharged without an established diagnosis: risk of mortality and readmission of nonspecific diagnoses compared to disease-specific diagnoses
Source: Scand J Trauma Resusc Emerg Med. 2024 Apr 19;32:32. doi: 10.1186/s13049-024-01191-4 (PMC11027222; doi:10.1186/s13049-024-01191-4)
Supplement: Supplementary file 1 — Supplementary Material 1 [file 13049_2024_1191_MOESM1_ESM.rtf]

Table S1. Hospital departments considered as Emergency Departments (ED) and used for inclusion of hospital courses.

The departments were identified extracting departments that included the Danish terms “akut”, “modtag”, “FAM”, “trauma” and “skade” (English: “acute”, “receive”, “joint emergency department”, “trauma”, and “injury”) from a list of all departments in the period. From there, we excluded departments traditionally outside of an ED setting, such as psychiatry, specialized units (Ophthalmology, Ear-nose-throat, among others) and ambulatories.
Departments are usually identified from a full 7-digit Sygehus-afdelingsklassifikationssystemet-code (SHAK-code, English: The classification system for hospitals and departments). To not exclude light misregistrations, we also considered 6-digit SHAK-codes—therefore, the same department can seem listed double below.


Hospital	Hospital code (c_sgh)	Department code (c_afd)	Name	SHAK-code	
Rigshospitalet	1301	28	Traumecenter og Akut Modtagelse, TC	130128	
	1301	288	Traumecenter og Akut Modtagelse TC, Visiteret skade	1301288	
	1301	32Q	Børnemodtagelsen, GGK	130132Q	
	1301	85X	Akutklinikken GLO	130185X	
Bispebjerg og Frederiksberg Hospitaler	1309	47	Akut modtageafd. AMA, Bispebjerg Hospital	130947	
	1309	470	Akutmodtagelse, Medicinsk Modtageafdeling	1309470	
	1309	478	Akutmodtagelse, Skadeafdeling	1309478	
	1309	47A	Akutklinik, FRH	130947A	
	1309	47D	Akutmodtagelse, Medicinsk Deldøgnsafdeling	130947D	
	1309	47G	Akutklinik, FRH	130947G	
	1309	695	Medicinsk Modtageafdeling, FRH	1309695	
	1309	698	Akutklinik, FRH	1309698	
Amager og Hvidovre Hospital	1330	168	Gyn.-obst. skadeafsnit	1330168	
	1330	19	Akut modtageafdeling, Hvidovre Hospital	133019	
	1330	190	Akut modtageafdeling	1330190	
	1330	324	Akutklinik Observation, AMH	1330324	
	1330	32A	Akutklinik, AMH	133032A	
	1330	604	Børnemodtagelsen, afd.	1330604	
	1330	62	Skadestue, Hvidovre Hospital	133062	
	1330	628	Skadestuen	1330628	
Amager Hospital	1351	40	Akut Modtagelse Overafdeling	135140	
	1351	400	Akut Modtagelse Observation	1351400	
	1351	408	Akutmodtagelse Skader	1351408	
Gentofte Hospital	1501	098	Akutklinik	1501098	
	1501	29	Modtagelsen overafd. W	150129	
	1501	290	Modtagelsen w	1501290	
Glostrup Hospital	1502	06X	Akutklinikken MSAK	150206X	
	1502	208	Skadestuen	1502208	
	1502	37	Modtagelsen overafd. 37	150237	
	1502	370	Modtagelsen	1502370	
	1502	57	SKADESTUE OVERAFDELING SKA.	150257	
	1502	578	SKADESTUE AFDELING SKA	1502578	
Herlev og Gentofte Hospital	1516	178	Skadestue	1516178	
	1516	36	Overafd. Akutmodtagelsen A	151636	
	1516	360	Akutmodtagelse A sengeafd.	1516360	
	1516	361	Akutklinik, sengeafd., GE	1516361	
	1516	367	Akutklinik, GE	1516367	
	1516	368	Skadestue	1516368	
	1516	378	Børne- og Ungemodtagelse	1516378	
	1516	438	AKUTKLINIK, GE	1516438	
Hospitalerne i Nordsjælland	2000	17	Akut overafdeling	200017	
	2000	170	HI, Akutafd., senge	2000170	
	2000	171	FS Akutklinik, senge	2000171	
	2000	177	SH Akutklinik, skadestue	2000177	
	2000	178	HI, Akutafd., skadestue	2000178	
	2000	179	FS Akutklinik, skadestue	2000179	
Region Sjællands Sygehusvæsen	3800	A08	ROS Med. Skade	3800A08	
	3800	D8	KOE Akutafdeling	3800D8	
	3800	D80	KOE Akut Afd.	3800D80	
	3800	D87	KOE Skade, Roskilde	3800D87	
	3800	D88	KOE Akut Afd., Skadestue	3800D88	
	3800	H9	HOL Akutafdeling	3800H9	
	3800	H90	HOL Akut Afd.	3800H90	
	3800	H97	HOL Skadeklinik, Nyk.Sj	3800H97	
	3800	H98	HOL Akut Afd., Skadestue	3800H98	
	3800	L9	KAL Akutafdeling	3800L9	
	3800	L98	KAL Skadeklinik	3800L98	
	3800	R8	SLA Akutafdeling	3800R8	
	3800	R80	SLA Akut Afd.	3800R80	
	3800	R87	SLA Skadestue, Næstved	3800R87	
	3800	R88	SLA Akut Afd., Skadestue	3800R88	
	3800	W4	NFS Akutafdeling	3800W4	
	3800	W40	NFS Akut Afd.	3800W40	
	3800	W46	NFS Skadeklinik, Nakskov	3800W46	
	3800	W48	NFS Akut Afd., Skadestue	3800W48	
Bornholms Hospital	4001	048	Skader	4001048	
	4001	10	Akutmodtagelse Overafdeling	400110	
	4001	100	Akutmodtagelse Observation	4001100	
	4001	108	Akutmodtagelsen	4001108	
OUH Odense Universitetshospital	4202	19	Skadestuen, Odense Universitetshospital	420219	
	4202	198	Od Skadestuen	4202198	
	4202	43	OUH FællesAkutModtag (Odense)	420243	
	4202	430	OUH FAM Medicinsk (Odense)	4202430	
	4202	434	OUH FAM A Nyremedicinsk (Odense)	4202434	
	4202	43A	OUH FAM Organkirurgi (Odense)	420243A	
	4202	43B	OUH FAM A Organkirurgi (Odense)	420243B	
	4202	43C	OUH FAM Reumatologi (Odense)	420243C	
	4202	43D	OUH FAM Kvindesygdomme (Odense)	420243D	
	4202	43E	OUH FAM A Endokrinologi (Odense)	420243E	
	4202	43G	OUH FAM Geriatrisk (Odense)	420243G	
	4202	43H	OUH FAM A Neurologi (Odense)	420243H	
	4202	43I	OUH FAM A Infektionsmedicin (Odense)	420243I	
	4202	43J	OUH FAM Lungemedicinsk (Odense)	420243J	
	4202	43K	OUH FAM A Karkirurgi (Odense)	420243K	
	4202	43L	OUH FAM Urinvejskirurgi (Odense)	420243L	
	4202	43M	OUH FAM Endokrinologi (Odense)	420243M	
	4202	43N	OUH FAM Neurologi (Odense)	420243N	
	4202	43O	OUH FAM Ortopædkirurgi (Odense)	420243O	
	4202	43P	OUH FAM A Med. Mave/Tarm (Odense)	420243P	
	4202	43Q	OUH FAM Infektionsmedicin (Odense)	420243Q	
	4202	43R	OUH FAM A Reumatologi (Odense)	420243R	
	4202	43S	OUH FAM Med. Mave-tarm (Odense)	420243S	
	4202	43T	OUH FAM Karkirurgi (Odense)	420243T	
	4202	43U	OUH FAM A Urinvejskirurgi (Odense)	420243U	
	4202	43V	OUH FAM A Lungemedicinsk (Odense)	420243V	
	4202	43W	OUH FAM A Kvindesygdomme (Odense)	420243W	
	4202	43X	OUH FAM A Ortopædkirurgi (Odense)	420243X	
	4202	43Y	OUH FAM Nyremedicinsk (Odense)	420243Y	
	4202	43Z	OUH FAM A Geriatrisk (Odense)	420243Z	
	4202	75	OUH Skadestuen (Svendborg)	420275	
	4202	759	OUH Skadestuen (Svendborg)	4202759	
	4202	77	OUH Fælles Akut Modtagelse (Svendborg)	420277	
	4202	770	OUH FAM Medicinsk (Svendborg)	4202770	
	4202	77A	OUH FAM Organkirurgi (Svendborg)	420277A	
	4202	77B	OUH FAM A Organkirurgi (Svendborg)	420277B	
	4202	77D	OUH FAM Kvindesygdomme (Svendborg)	420277D	
	4202	77L	OUH FAM Urinvejskirurgi (Svendborg)	420277L	
	4202	77O	OUH FAM Ortopædkirurgi (Svendborg)	420277O	
	4202	77U	OUH FAM A Urinvejskirurgi (Svendborg)	420277U	
	4202	77W	OUH FAM A Kvindesygdomme (Svendborg)	420277W	
	4202	77X	OUH FAM A Ortopædkirurgi (Svendborg)	420277X	
Sygehus Sønderjylland	5000	10	SHS Fælles Akut Modtagelse (FAM)	500010	
	5000	100	SHS FAM Sengeafsnit (Aabenraa)	5000100	
	5000	108	SHS Skadestue (Aabenraa)	5000108	
	5000	10C	SHS FAM Hjertesygdomme Sengeafsnit (Aabenraa)	500010C	
	5000	10G	SHS FAM Kvindesygdomme Sengeafsnit (Aabenraa)	500010G	
	5000	10K	SHS FAM Kirurgi Sengeafsnit (Aabenraa)	500010K	
	5000	10M	SHS FAM Medicinske Sygdomme Sengeafsnit (Aabenraa)	500010M	
	5000	10N	SHS FAM Hjerne- og Nervesygdomme Sengeafsnit (Aaben	500010N	
	5000	10O	SHS FAM Ortopædkirurgi Sengeafsnit (Aabenraa)	500010O	
	5000	10S	SHS Skadeklinik (Sønderborg)	500010S	
	5000	10T	SHS Skadeklinik (Tønder)	500010T	
	5000	10U	SHS FAM Urinvejskirurgi Sengeafsnit (Aabenraa)	500010U	
	5000	63	SHS Medicinsk Modtagelse	500063	
	5000	630	SHS Medicinsk Modtagelse Sengeafsnit (Sønderborg)	5000630	
Sydvestjysk Sygehus	5501	02	Akut område Esbjerg	550102	
	5501	028	SVS Skadestue (Esbjerg)	5501028	
	5501	22	SVS Afdeling for Fælles Akut Modtagelse	550122	
	5501	22A	SVS FAM Parenkymkirurgisk afsnit (Esbjerg)	550122A	
	5501	22B	SVS FAM Kardiologisk afsnit (Esbjerg)	550122B	
	5501	22D	SVS FAM Gynækologisk afsnit (Esbjerg)	550122D	
	5501	22F	SVS FAM Øre-Næse-Hals afsnit (Esbjerg)	550122F	
	5501	22H	SVS FAM Børne- og ungeafsnit (Esbjerg)	550122H	
	5501	22N	SVS FAM Neurologisk afsnit (Esbjerg)	550122N	
	5501	22O	SVS FAM Ortopædkirurgisk afsnit (Esbjerg)	550122O	
	5501	22W	SVS FAM Medicinsk afsnit (Esbjerg)	550122W	
	5501	42	Akut område Grindsted	550142	
	5501	52	Akut område Brørup	550152	
Regionshospitalet Horsens	6006	18	Akutafdelingen, Overafdeling - RHH	600618	
	6006	181	Akutafdelingen, Sengeafdeling - RHH	6006181	
	6006	188	Akutafdelingen, Skadestuen - RHH	6006188	
	6006	189	Akutafdelingen, Akutklinik - RHH	6006189	
Kolding Sygehus	6007	21	SLB Akutafdelingen (Kolding)	600721	
	6007	210	SLB Fælles Akut Modtagelse (Kolding)	6007210	
	6007	211	SLB FAM A Børnesygdomme (Kolding)	6007211	
	6007	212	SLB FAM A Hjerne- og nervesygdomme (Kolding)	6007212	
	6007	213	SLB FAM A Hjertesygdomme (Kolding)	6007213	
	6007	214	SLB FAM A Karkirurgi (Kolding)	6007214	
	6007	215	SLB FAM A Kvindesygdomme (Kolding)	6007215	
	6007	216	SLB FAM A Medicinske Sygdomme (Kolding)	6007216	
	6007	218	SLB FAM A Ortopædkirurgi (Kolding)	6007218	
	6007	21A	SLB FAM Organkirurgi (Kolding)	600721A	
	6007	21B	SLB FAM Hjertesygdomme (Kolding)	600721B	
	6007	21D	SLB FAM Kvindesygdomme (Kolding)	600721D	
	6007	21F	SLB FAM Akutafdeling (Kolding)	600721F	
	6007	21H	SLB FAM Børnesygdomme (Kolding)	600721H	
	6007	21N	SLB FAM Hjerne- og Nervesygdomme (Kolding)	600721N	
	6007	21O	SLB FAM Ortopædkirurgi (Kolding)	600721O	
	6007	21T	SLB FAM Karkirurgi (Kolding)	600721T	
	6007	21W	SLB FAM Medicinske Sygdomme (Kolding)	600721W	
Vejle Sygehus	6008	053	SLB Akut visitations Afsnit (Vejle)	6008053	
	6008	208	SLB Skadestuen (Vejle)	6008208	
	6008	20F	SLB Skadestue (Vejle)	600820F	
	6008	217	SLB FAM A Organkirurgi (Kolding)	6008217	
	6008	38	SLB Skadestue (Vejle)	600838	
	6008	38B	SLB Hjertemedicinsk Skadestue (Vejle)	600838B	
	6008	38E	SLB Øjen Skadestue (Vejle)	600838E	
	6008	38F	SLB Øre-Næse-Hals Skadestue (Vejle)	600838F	
	6008	38L	SLB Urinvejskirurgisk Skadestue (Vejle)	600838L	
	6008	38O	SLB Ortopædkirurgisk Skadestue (Vejle)	600838O	
	6008	38W	SLB Medicinsk Skadestue (Vejle)	600838W	
Aarhus Universitetshospital	6620	188	Skadestuen Ortopædkirurgisk Afdeling	6620188	
	6620	18U	Ortopædkirurgi Akut Stamafdeling	662018U	
	6620	18V	Ortopædkirurgi - Akut Klinik	662018V	
	6620	248	Børn og Unge Afsnit for Akut Sygdom	6620248	
	6620	37	Akutafdeling Overafdeling	662037	
	6620	371	Akutafdeling Akutafsnit	6620371	
	6620	373	Akutafdeling Akut Børn	6620373	
	6620	375	Akutafdeling Traumecenter	6620375	
	6620	376	Akutafdeling Akut COVID Klinik	6620376	
	6620	377	Akutafdeling Klinik	6620377	
	6620	378	Akutafdeling Skadestue	6620378	
Hospitalsenhed Midt	6630	20	Akutafdelingen	663020	
	6630	201	Akutafsnit	6630201	
	6630	209	Akutafdeling HE Midt	6630209	
	6630	20A	Skadestue	663020A	
	6630	20B	Akutklinik Silkeborg	663020B	
	6630	20C	Akutklinik Skive	663020C	
Regionshospitalet Gødstrup	6640	20	Akutafdelingen	664020	
	6640	20B	Akutmodtagelse Gødstrup	664020B	
	6640	20D	Akutklinik Center for Sundhed Holstebro	664020D	
	6640	20E	Akutklinik Sundhedshus Ringkøbing	664020E	
	6640	20F	Akutklinik Lemvig Sundhedshus - RHG	664020F	
	6640	24B	Akutmodtagelse for Børn og Unge	664024B	
	6640	40B	Neurologisk Akutklinik Gødstrup	664040B	
Hospitalsenheden Vest	6650	20	Akutafdeling	665020	
	6650	202	Akut senge Herning	6650202	
	6650	208	Skadestue Holstebro	6650208	
	6650	209	Skadestue Herning	6650209	
	6650	20X	Akutklinik Ringkøbing	665020X	
Regionshospitalet Randers	7005	05E	Akut Hjerteklinik - Randers	700505E	
	7005	40	Akutafdelingen - Randers	700540	
	7005	401	Akutafdelingen - Randers	7005401	
	7005	405	Akutklinikken - Grenaa	7005405	
	7005	406	Fælles Akutmodtagelse - Randers	7005406	
	7005	408	Skadestuen - Randers	7005408	
	7005	409	Akut Dagklinik - Randers	7005409	
Samsø Sundheds- og Akuthus	7013	016	Akutklinik Samsø	7013016	
	7013	018	Skadestuen Afdeling Samsø	7013018	
Aalborg Universitetshospital, Thisted	7603	46	Thy Akutmodtagelsen	760346	
	7603	461	Thy Akut Modtageafdeling	7603461	
	7603	468	Thy Skadestue	7603468	
	7603	46V	Thy Akut Visitation	760346V	
Aalborg Universitetshospital	8001	08A	Alb Gynækologisk Akut Område	800108A	
	8001	46	Alb Akut- og Traumecenter	800146	
	8001	461	Alb Akut Modtageafdeling	8001461	
	8001	468	Alb Skade-Modtageafdeling	8001468	
	8001	538	ON Skadestuer	8001538	
	8001	661	Hob Akut Medicinsk Modtageafdeling	8001661	
	8001	668	Hob Småskadeklinik Afdeling	8001668	
Regionshospital Nordjylland Ven	8003	46	Ven Akutmodtagelsen FAM	800346	
	8003	461	Hjr Akut Modtageafdeling	8003461	
	8003	464	Hjr Akut Pandemi Senge	8003464	
	8003	468	Hjr Skadestue	8003468	
