# Supplementary material for: Acute patients discharged without an established diagnosis: risk of mortality and readmission of nonspecific diagnoses compared to disease-specific diagnoses
Source: Scand J Trauma Resusc Emerg Med. 2024 Apr 19;32:32. doi: 10.1186/s13049-024-01191-4 (PMC11027222; doi:10.1186/s13049-024-01191-4)
Supplement: Supplementary file 2 — Supplementary Material 2 [file 13049_2024_1191_MOESM2_ESM.rtf]

Table S2. Review of the R- and Z-ICD10 primary diagnoses used among all contacts considered for inclusion in the study period. These diagnoses were either considered unspecific, disease-specific or excluded.
Unspecific diagnoses were further reviewed to form clinical subgroups of patients.

Group	ICD-10 code	
Abnormal heart rhythm	'DR000'-'DR001', 'DR008', 'DR008A'	
Palpitations	'DR002'	
Other cardiopulmonary symptom or finding	'DR010'-'DR012A', 'DR030'-'DR031', 'DR098'-'DR098F'	
Epistaxis and oropharyngeal bleeding	'DR040'-'DR041'	
Bleeding from airways	'DR042'-'DR049'	
Coughing	'DR059', 'DR093'-'DR093B'	
Abnormal breathing	'DR060'-'DR064', 'DR090', 'DR068'-'DR068D'	
Sore throat, hoarseness, and altered voice	'DR070', 'DR490'-'DR498', 'DR498A'-'DR498B'	
Chest pain	'DR071'-'DR0749Z'	
Abdominal pain	'DR10'-'DR109Z', 'DR193', 'DR198E ', 'DR198F'	
Nausea or vomiting	'DR119'-'DR119C'	
Dysphagia and eating difficulties	'DR139', 'DR633'-'DR633A', 'DR638'	
Abnormal weight loss and cachexia	'DR634', 'DR649'	
Other gastrointestinal symptom or finding	'DR129', 'DR149'-'DR162B', 'DR190'-'DR192ZZ',
'DR193A'-'DR195',
'DR198'-'DR198D', 'DR198G', 'DR630'	
Icterus	'DR179'	
Ascites	'DR189'	
Abnormal findings of the skin	'DR219'-'DR238B'	
Symptoms and signs involving the nervous and musculoskeletal systems	'DR200'-'DR208A', 'DR250'-'DR251', 'DR252C', 'DR253'-'DR260', 'DR262', 'DR268', 'DR268B'-'DR268F', 'DR270'-'DR294', 'DR298', 'DR298A',
	'DR470'-'DR488B'	
Tendency to fall	'DR296'	
Symptoms regarding urination and urinary tract	'DR300'-'DR391E', 'DR398'-'DR398E'	
Altered mental status and amnesia	'DR400'-'DR410', 'DR411'-'DR418', 'DR464', 'DR549', 'DR549A'	
Vertigo	'DR429'	
Psychiatric, mood, and psychomotoric symptoms	'DR298B', 'DR440'-'DR463', 'DR465'-'DR468', 'DR418'	
Fever	'DR50'-'DR509Z'	
Headache, unspecified	'DR519'	
Acute pain, unspecified	'DR520', 'DR529'	
Chronic pain, unspecified	'DR521'-'DR522A', 'DR522D'-'DR522E'	
Pain in genital area	'DR522B', 'DR522C'	
Malaise or fatigue	'DR53'-'DR539Z', 'DR549B'	
Fainting	'DR55'-'DR559Z'	
Seizures	'DR252'-'DR252B', 'DR560'-'DR568E', 'DR568G'	
Bleeding, unspecified	'DR589'	
Enlarged lymph node	'DR590'-'DR599'	
Oedema	'DR600'-'DR609'	
Abnormal biochemical result	'DR700'-'DR779', 'DR790'-'DR899'	
Abnormal biochemical result (exogenous substance)	'DR780'-'DR789'	
Abnormal radiology or clinical physiology examination	'DR900'-'DR949'	
Moved to the disease-specific group	'DR029' (Gangrene, not elsewhere classified)
'DR091' (Pleuritis, not classified elsewhere)	
'DR092' (Cardiopulmonary failure)
'DR392', 'DR392A', 'DR392B' (Uremia)
'DR568F', 'DR570', 'DR571', 'DR572', 'DR578', 'DR578A', 'DR579' (Shock)
	
Exclude	'DR67'-'DR678' (Barthel index)	
Other R-diagnoses	Remaining R-diagnoses	
Observation for suspected cancer	'DZ031'-'DZ031Z', 'DZ038E'	
Observation for suspected nervous system disorder, unspecified	'DZ033'	
Observation for suspected epilepsy	'DZ033A'	
Observation for concussion	'DZ033D'	
Observation for stroke	'DZ033E'	
Other Z03 diagnoses	'DZ030', 'DZ032', 'DZ032A', 'DZ033B', 'DZ033C', 'DZ033F'-'DZ034', 'DZ036'-'DZ037', 'DZ038B', 'DZ038D', 'DZ038F'-'DZ038H', 'DZ038K', 'DZ038KA'	
Observation for suspected myocardial infarction	'DZ034'	
Observation for other suspected cardiovascular diseases	'DZ035', 'DZ035C'-'DZ035EA'	
Observation for suspected arrythmia	'DZ035A'	
Observation for suspected urinary tract infection	'DZ038A'	
Observation for suspected allergic condition	'DZ038C'	
Observation for suspected smoke inhalation	'DZ038J'	
Observation for unspecified disease or condition	'DZ038', 'DZ039'	
Excluded (injuries)	'DZ041'-'DZ045' (Contact due to injury or damage)
'DZ478A', 'DZ478B' (Contact for adjustment of plaster or bandage)
'DZ230'-'DZ279' (Contact with the purpose of vaccination)
	
Exclude (Pregnancy and birth)	'DZ3'-'DZ39311', 'DZ038M', 'DZ038O', 'DZ640'	
Move to disease group	'DZ895'-'DZ908A', 'DZ930'-'DZ988', 'DZ609' (Conditions following surgeries (amputations, implants, pacemakers, transplants, stomas etc.))
'DZ219'-'DZ229' (HIV-infection)
'DZ721'-'DZ7222' (Problem with alcohol or substance use)
'DZ200'-'DZ209' (Exposed of infectious disease)
'DZ291'-'DZ292A' (Contact with the purpose of prophylactic treatment)	
Administrative diagnoses (excluded)	Remaining Z-diagnoses	
