# Supplementary material for: Acute patients discharged without an established diagnosis: risk of mortality and readmission of nonspecific diagnoses compared to disease-specific diagnoses
Source: Scand J Trauma Resusc Emerg Med. 2024 Apr 19;32:32. doi: 10.1186/s13049-024-01191-4 (PMC11027222; doi:10.1186/s13049-024-01191-4)
Supplement: Supplementary file 3 — Supplementary Material 3 [file 13049_2024_1191_MOESM3_ESM.docx]

**Table S3**. Cause-specific hazard ratios of mortality and readmission within 30 for patients with unspecific compared to disease-specific discharge diagnoses, unadjusted and with gradual adjustments. Stratified into short and longer hospital courses

|  | ***Short hospital course (3 - <12 hours)*** | | ***Longer hospital course (12-168 hours)*** | |
| --- | --- | --- | --- | --- |
|  | **Mortality** | **Readmission**^†^ | **Mortality** | **Readmission**^†^ |
| Unadjusted | 0.79 (0.68-0.92) | 0.87 (0.83-0.91) | 0.62 (0.55-0.68) | 0.80 (0.77-0.83) |
| Model 1 | 0.78 (0.67-0.90) | 0.87 (0.83-0.91) | 0.68 (0.61-0.75) | 0.83 (0.80-0.87) |
| Model 2 | 0.78 (0.67-0.90) | 0.87 (0.83-0.91) | 0.68 (0.61-0.76) | 0.84 (0.80-0.87) |
| Model 3 | 0.80 (0.69-0.93) | 0.88 (0.84-0.92) | 0.71 (0.64-0.79) | 0.86 (0.82-0.89) |
| Model 4 | 0.81 (0.69-0.94) | 0.88 (0.84-0.92) | 0.82 (0.74-0.92) | 0.89 (0.85-0.93) |
| Model 5 | 0.97 (0.83-1.13) | 0.94 (0.90-0.98) | 0.94 (0.85-1.05) | 0.95 (0.91-0.99) |

† Death before readmission was considered a competing event

Model 1: Adjusted for age and sex

Model 2: Adjusted for model 1 variables + sociodemograpchis (educational level, employment, civil status, cohabitation status, income, immigration status, and country of origin)

Model 3: Adjusted for model 2 variables + M3 comorbidity score

Model 4: Adjusted for model 3 variables + administrative information (time and day of arrival, time and day of discharge, and length of stay)

Model 5: Adjusted for model 4 variables + laboratory information (whether at least 5 blood tests were analyzed among the 16 most frequent analyzes and number of abnormal results among these)
