# Supplementary material for: Acute patients discharged without an established diagnosis: risk of mortality and readmission of nonspecific diagnoses compared to disease-specific diagnoses
Source: Scand J Trauma Resusc Emerg Med. 2024 Apr 19;32:32. doi: 10.1186/s13049-024-01191-4 (PMC11027222; doi:10.1186/s13049-024-01191-4)
Supplement: Supplementary file 4 — Supplementary Material 4 [file 13049_2024_1191_MOESM4_ESM.rtf]

Table S4. Full list of clinical subgroups of patients with unspecific diagnoses and corresponding characteristics and outcomes.

ICD-10 chapter	Group	n	Age, mean	Female, %	M3 Score, mean	LOS (hours), mean	Risk of mortality within 30 days, % (95% CI)	Risk of readmission within 30 days, % (95% CI)	
All	All R and Z03	72950	57.3	53.6%	0.55	18	1.0% (0.9-1.1%)	8.6% (8.4-8.9%)	
R	All	58924	56.9	54.1%	0.55	17.6	0.9% (0.9-1.0%)	8.8% (8.6-9.0%)	
R	Chest pain	9599	55.1	50.8%	0.45	11.1	0.2% (0.1-0.3%)	4.5% (4.2-4.9%)	
R	Abnormal heart rhythm	428	65.3	50.9%	0.64	31	1.6% (0.8-3.3%)	8.9% (6.5-12.1%)	
R	Palpitations	1299	50.9	63.8%	0.33	7.8	Censored, few events	3.5% (2.7-4.7%)	
R	Other cardiopulmonary symptom or finding	97	64.8	66.0%	0.44	14.9	Censored, few events	12.3% (7.0-21.6%)	
R	Coughing	688	56.3	56.4%	0.56	10.2	Censored, few events	8.3% (6.5-10.5%)	
R	Abnormal breathing	3788	64.2	55.9%	0.89	22.3	2.7% (2.3-3.3%)	12.5% (11.5-13.6%)	
R	Sore throat, hoarseness, and altered voice	59	43.5	59.3%	0.5	7.1	Censored, few events	Censored, few events	
R	Epistaxis and oropharyngeal bleeding	1160	69.6	45.6%	0.73	11.2	1.2% (0.7-2.0%)	10.6% (9.2-12.3%)	
R	Bleeding from airways	210	61.9	39.0%	0.75	23.9	Censored, few events	7.6% (5.1-11.3%)	
R	Abdominal pain	12462	46.9	64.3%	0.41	15.2	0.5% (0.4-0.6%)	9.7% (9.2-10.1%)	
R	Nausea or vomiting	998	56.3	61.1%	0.68	21.7	1.7% (1.1-2.7%)	13.7% (11.6-16.1%)	
R	Dysphagia and eating difficulties	383	67.2	39.2%	1.07	24.5	4.7% (2.9-7.6%)	15.1% (11.8-19.3%)	
R	Icterus	126	64.2	34.9%	0.64	59.5	7.1% (3.5-14.3%)	27.6% (20.5-37.0%)	
R	Ascites	139	68.5	40.3%	1.6	50.9	15.0% (10.3-21.9%)	33.5% (26.6-42.0%)	
R	Other gastrointestinal symptom or finding	198	56.9	46.5%	0.51	20	Censored, few events	13.6% (9.4-19.5%)	
R	Symptoms and signs involving the nervous and musculoskeletal systems	2421	54.9	58.3%	0.49	26.7	0.2% (0.1-0.6%)	7.4% (6.4-8.5%)	
R	Tendency to fall	958	80.8	54.0%	1.03	48.3	2.3% (1.5-3.5%)	16.0% (14.1-18.2%)	
R	Seizures	1122	51.7	39.3%	0.79	22.1	1.1% (0.6-1.8%)	9.1% (7.8-10.5%)	
R	Vertigo	2921	64.3	59.3%	0.48	18.3	Censored, few events	6.0% (5.2-7.0%)	
R	Headache, unspecified	2578	47.6	62.6%	0.34	13.7	Censored, few events	7.5% (6.5-8.7%)	
R	Altered mental status and amnesia	628	76.3	54.1%	0.96	34.9	3.7% (2.5-5.4%)	15.5% (12.9-18.7%)	
R	Psychiatric, mood, and psychomotoric symptoms	135	57.1	45.2%	0.56	10	Censored, few events	11.0% (7.0-17.5%)	
R	Symptoms regarding urination and urinary tract	2495	71.6	15.3%	0.77	26.2	2.1% (1.6-2.8%)	16.1% (14.7-17.7%)	
R	Pain in genital area	85	41.9	10.6%	0.33	7.5	Censored, few events	Censored, few events	
R	Abnormal findings of the skin	682	57	53.2%	0.47	13.1	Censored, few events	5.3% (3.9-7.1%)	
R	Malaise or fatigue	2214	63.1	56.1%	0.64	10.8	1.4% (1.0-1.9%)	8.4% (7.5-9.5%)	
R	Fainting	4708	62	49.2%	0.52	20.5	0.4% (0.3-0.6%)	5.9% (5.3-6.5%)	
R	Fever	1046	53.9	43.0%	0.64	32.1	1.3% (0.8-2.4%)	14.6% (12.9-16.7%)	
R	Abnormal weight loss and cachexia	72	67.8	40.3%	0.73	59.2	9.6% (4.4-21.1%)	20.6% (14.0-30.3%)	
R	Acute pain, unspecified	3460	56.5	59.0%	0.51	10.6	0.8% (0.6-1.2%)	7.8% (6.9-8.8%)	
R	Chronic pain, unspecified	120	62.5	55.8%	1.06	26.8	Censored, few events	17.4% (12.5-24.4%)	
R	Bleeding, unspecified	157	67.4	44.6%	0.96	17.2	6.3% (3.4-11.8%)	13.3% (8.7-20.5%)	
R	Enlarged lymph node	35	44.5	45.7%	0.33	11	Censored, few events	Censored, few events	
R	Oedema	628	66.7	54.1%	0.69	16.1	1.7% (0.9-3.3%)	10.0% (7.9-12.8%)	
R	Abnormal biochemical result	410	59.2	42.7%	0.89	37	2.2% (1.1-4.3%)	14.1% (11.0-18.0%)	
R	Abnormal biochemical result (exogenous substance)	264	44.7	42.0%	0.44	10.7	Censored, few events	4.9% (3.0-8.1%)	
R	Abnormal radiology or clinical physiology examination	42	62.8	42.9%	0.57	57.3	Censored, few events	11.8% (5.1-27.3%)	
R	Other R-diagnoses	109	63.3	40.4%	0.69	19.9	6.4% (3.2-12.8%)	14.6% (10.1-21.0%)	
Z03	All	14026	59.5	51.3%	0.56	19.7	1.2% (1.0-1.4%)	8.1% (7.6-8.6%)	
Z03	Observation for suspected myocardial infarction	2656	61.7	47.6%	0.58	21.1	0.3% (0.1-0.5%)	5.3% (4.6-6.1%)	
Z03	Observation for suspected arythmia	474	61	48.3%	0.51	26.2	Censored, few events	5.9% (4.1-8.5%)	
Z03	Observation for suspected cancer	833	69.3	43.0%	0.6	58.8	8.1% (6.5-10.2%)	22.6% (20.2-25.3%)	
Z03	Observation for suspected epilepsy	108	54.4	46.3%	0.61	26.6	Censored, few events	10.1% (5.7-17.8%)	
Z03	Observation for concussion	1526	59.3	49.4%	0.61	10.2	1.0% (0.7-1.7%)	6.7% (5.6-8.0%)	
Z03	Observation for stroke	657	66.1	55.3%	0.6	30.5	0.8% (0.3-1.8%)	8.0% (6.3-10.3%)	
Z03	Observation for suspected nervous system disorder, unspecified	747	56.2	57.0%	0.49	21.2	0.8% (0.4-1.8%)	6.9% (5.5-8.8%)	
Z03	Observation for suspected urinary tract infection	191	67.3	66.0%	0.87	30	2.6% (1.0-6.6%)	14.0% (10.4-18.9%)	
Z03	Observation for suspected allergic condition	87	52	65.5%	0.44	11.9	Censored, few events	Censored, few events	
Z03	Observation for suspected smoke inhalation	139	44	38.1%	0.3	8.3	Censored, few events	Censored, few events	
Z03	Observation for unspecified disease or condition	4973	56.5	53.3%	0.55	12.3	1.0% (0.8-1.3%)	8.4% (7.6-9.3%)	
Z03	Other Z03 diagnoses	200	56	55.5%	0.48	31.8	Censored, few events	9.0% (5.9-13.6%)	

Cells with < 5 events are censored
Abbreviations: CI = confidence interval; M3 Score = M3 comorbidity index score; LOS = length-of-stay
